# Diethyl Succinate Modulates Microglial Polarization and Activation by Reducing Mitochondrial Fission and Cellular ROS

**DOI:** 10.3390/metabo11120854

**Published:** 2021-12-08

**Authors:** Lixiang Wang, Yanli Zhang, Magdalena Kiprowska, Yuqi Guo, Ken Yamamoto, Xin Li

**Affiliations:** 1 Department of Molecular Pathobiology, New York University College of Dentistry, 345 E. 24th Street, Room 901D, New York, NY 10010, USA; ourika0211@med.kurume-u.ac.jp (L.W.); zyl2080@126.com (Y.Z.); mkiprowskak2@gmail.com (M.K.); yg701@nyu.edu (Y.G.); 2 Department of Medical Biochemistry, Kurume University School of Medicine, Kurume 830-0011, Japan; yamamoto_ken@med.kurume-u.ac.jp; 3 Perlmutter Cancer Institute, New York University Grossman School of Medicine, New York, NY 10016, USA; 4 Department of Urology, New York University Grossman School of Medicine, New York, NY 10016, USA

**Keywords:** succinate, microglia, mitochondrial fission, LPS, dynamin-related protein

## Abstract

Succinate is a metabolite in the tricarboxylic acid cycle (TCA) which plays a central role in mitochondrial activity. Excess succinate is known to be transported out of the cytosol, where it activates a succinate receptor (SUCNR1) to enhance inflammation through macrophages in various contexts. In addition, the intracellular role of succinate beyond an intermediate metabolite and prior to its extracellular release is also important to the polarization of macrophages. However, the role of succinate in microglial cells has not been characterized. Lipopolysaccharide (LPS) stimulates the elevation of intracellular succinate levels. To reveal the function of intracellular succinate associated with LPS-stimulated inflammatory response in microglial cells, we assessed the levels of ROS, cytokine production and mitochondrial fission in the primary microglia pretreated with cell-permeable diethyl succinate mimicking increased intracellular succinate. Our results suggest that elevated intracellular succinate exerts a protective role in the primary microglia by preventing their conversion into the pro-inflammatory M1 phenotype induced by LPS. This protective effect is SUCNR1-independent and mediated by reduced mitochondrial fission and cellular ROS production.

## 1. Introduction

Succinate is best known as a metabolic intermediate in the tricarboxylic acid cycle (TCA), in which it is the substrate for mitochondrial complex II–succinate dehydrogenase (SDH) and as such an important component of oxidative phosphorylation. It is at a crossroads of many cellular metabolic pathways such as macrophage-specific metabolic pathway-generating itaconate, the metabolism of branched-chain amino acids, as well as heme synthesis, ketone body utilization and the gamma aminobutyric acid (GABA) shunt [1,2,3,4]. Emerging evidence has suggested succinate’s role as a signaling transmitter both intracellularly and extracellularly. Produced in the mitochondria, where it is involved in cellular metabolism, succinate accumulates in response to certain pathological stimuli and gets transported to the cytosol, where it participates in signal transduction pathways. Once succinate is transported outside of the cell, it acts extracellularly through succinate receptor 1 (SUCNR1) [5]. The presence of SUCNR1 has been confirmed in a wide range of tissues such as liver, retina, kidney, blood cell and adipose tissue [5,6]. Existing reports from our group [7] and others [8,9,10,11,12,13,14,15] suggest a pro-inflammatory response upon SUCNR1 activation in various tissues. The fact that the succinate receptor is expressed in different types of immune cells indicates SUCNR1 activation could be a common reaction during immune responses. SUCNR1 is expressed in macrophages, microglia as well as in human immature monocyte-derived dendritic cells (iMoDCs) [16]. In immature dendritic cells, succinate acts through transiently expressed SUCNR1 and in synergy with Toll-like receptor TLR4 leads to enhanced expression of pro-inflammatory cytokines [16]. Additionally, succinate triggers a migratory response in iMoDCs in a dose-dependent manner irrespective of TLR4 activation [16]. Other studies have also implicated SUCNR1’s role in cellular motility. For example, Ko et al. showed that human mesenchymal stem cell (hMSC) migration in a wound repair model is the result of mitochondrial fission initiated by succinate signaling through SUCNR1. As a consequence of increased mitochondrial fragmentation, the levels of ROS increase, F-actin is formed, and hMSC migration finally occurs [17]. Yet another study showed that in cardiomyocytes, an extracellular succinate-dependent increase in mitochondrial fission resulted in irreversible mitochondrial impairment and subsequent cell death [18]. These studies have successfully linked succinate and SUCNR1 activation to several critical physiological and cellular events (e.g., inflammatory responses, cellular mobility and mitochondrial dynamics).

In contrast, the intracellular role of succinate seems more complicated and requires further study. Accumulated succinate has been showed to inhibit the prolyl-hydroxylase domain (PHD), leading to stabilization of hypoxia inducible factor 1 (HIF-1) and activation of a range of target genes, including pro-inflammatory cytokine-IL-1β [16,19]. Importantly, Tannahill et al. [15] showed that succinate accumulation occurs in LPS-treated macrophages, and Chouchani et al. [20] discovered that succinate accumulation occurs in a response to ischemia in a range of tissues, and it leads to the production of high levels of ROS. These data seem to also suggest that the intracellular succinate acts as a mediator of inflammation. Alternatively, the association of succinate accumulation with these inflammatory events could indicate that the intracellular succinate accumulation is a negative feedback reaction in response to LPS and may counteract the intracellular effects induced by LPS. It is plausible failure of this attempt that may trigger succinate’s transportation out of the cytosol. Subsequently, the extracellular succinate leads to activation of SUCNR1 and enhanced inflammatory responses.

It has been shown that LPS treatment of the microglia increases succinate levels, switches metabolism to glycolysis and causes increased mitochondrial fission [21,22]. Interestingly, a number of studies indicated mitochondrial fission in the microglial models of inflammation, starting a debate about the role of excessive mitochondrial fragmentation in the activation of microglia [21,22]. Microglia, along with astrocytes, are involved in maintaining an optimal microenvironment for the neurons [23]. Interestingly, microglia are SUCNR1-expressing resident immune cells of the central nervous system (CNS). Under physiological conditions, they are highly ramified and use their processes to survey the CNS tissue for any debris. However, in response to a pathological stimulus, such as LPS or hypoxia, they become activated. Microglial activation is a process associated with altered metabolism, the secretion of pro-inflammatory cytokines and changing morphologies into less ramified and rounder cells. Fragmented mitochondria have been associated with molecular events implicated in microglial activation [22,24,25]. LPS treatment of BV-2 microglia was shown to elicit expression of pro-inflammatory cytokines, ROS, MAPK activation and NFκb translocation to the nucleus. These events are mitochondrial fission- dependent, as treatment with a fission inhibitor abrogates them [22]. The role of increased levels of intracellular succinate in microglial activation, if any, is not yet known. Our study attempts to bridge the gap in understanding the mechanism and role of intracellular succinate on mitochondrial morphology and microglial activation. Our aim in the current study was to dissect the effects of intracellular and extracellular succinate signaling on microglial functional phenotypes in response to LPS-induced inflammation. We used diethyl succinate, a cell-permeable form of succinate, to investigate the intracellular pathways and disodium succinate, an SUCNR1 ligand, to study the extracellular pathway. We showed that intracellular succinate exerted protective activity on LPS-activated microglia by reducing their M1 pro-inflammatory population. We found that these effects were likely a result of reduced mitochondrial fission due to increased phosphorylation of dynamin-related protein (DRP1) at Ser637 and associated with ROS reduction that prevented morphological transformation of the microglia into M1. Disodium succinate, the SUCNR1 ligand, had either no or a slightly opposing effect to the effects of diethyl succinate. Overall, our study highlights the versatility of succinate in terms of its intracellular and extracellular signaling in various inflammation-related contexts.

## 2. Results

### 2.1. Diethyl Succinate Decreased the M1 Population in the Primary Microglial Cells

**Figure 1:**
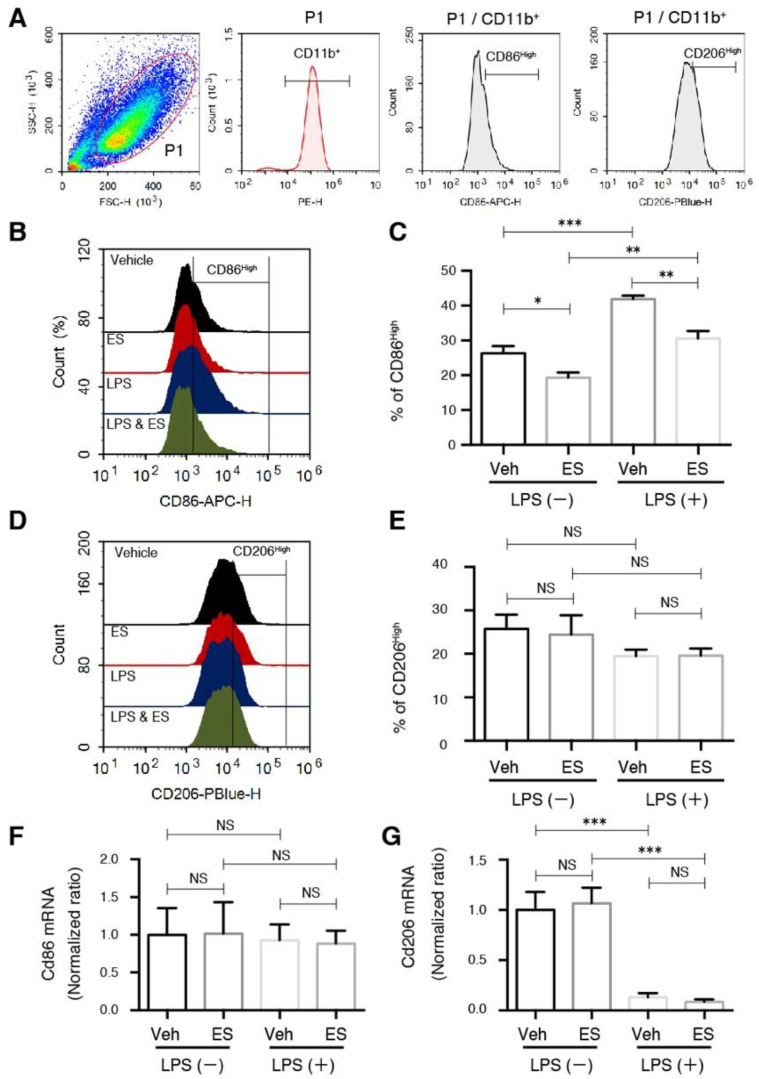
Diethyl succinate decreased the M1 population in primary microglial cells. Primary microglial cells were pretreated with or without diethyl succinate (ES: 5 mM) for 3 h before being stimulated with vehicle (phosphate-buffered saline (PBS)) or lipopolysaccharide (LPS) at 100 ng/mL for 24 h. Cells were washed and analyzed for flow cytometry (**A**–**E**) and real-time polymerase chain reaction (qPCR) results (**F**,**G**). (**A**) Gating strategy and percentage analysis of the M1 (CD11b^+^ CD86^High^) and M2 (CD11b^+^ CD206^High^) primary microglial cells. (**B**,**C**) Representative overlay image and quantification analysis of M1 surface marker. (**D**,**E**) Representative overlay image and quantification analysis of M2 surface marker. (**F**,**G**) The gene expression levels of *Cd86* and *Cd206* were determined by quantitative qPCR analysis and normalized to β-actin expression in each sample. Gene expression levels in each group normalized to the vehicle-treated group are shown (*n* = 3). Data represent mean  ±  standard error. * *p* < 0.05. ** *p* < 0.01. *** *p* < 0.001.

The activated M1 and M2 primary microglial cells were identified by the presence of cell surface markers CD11b^+^ CD86^High^ and CD11b^+^ CD206^High^, respectively (Figure 1A). We found that the cell-permeable diethyl succinate (ES) significantly decreased the pro-inflammatory M1 microglial population in both the lipopolysaccharide (LPS)-treated and untreated cells (Figure 1B,C). The population of M2 polarized microglial cells was not changed (Figure 1D,E). In contrast, disodium succinate (SS), which is not cell-permeable, only had slightly increased levels of CD11b^+^ CD86^High^-positive cells in the LPS-treated microglia, and this change was not significant (Supplementary Materials, Figure S1A,B). Similarly to diethyl succinate, disodium succinate had no effect on CD11b^+^ CD206^High^ in either the LPS-treated or untreated cells (Figure S1C,D).

The mRNA levels of the Cd86 and Cd206 cell markers were unchanged, indicating that the decrease in the M1 population was unlikely to be regulated through transcription reduction (Figure 1F,G). The significant drop in CD206 expression after (LPS) treatment is a universal response exhibited by microglia exposed to an inflammatory stimulus [26], and it was not affected by the ES.

### 2.2. Diethyl Succinate Reduced ROS Production in the Primary Microglial Cells

**Figure 2:**
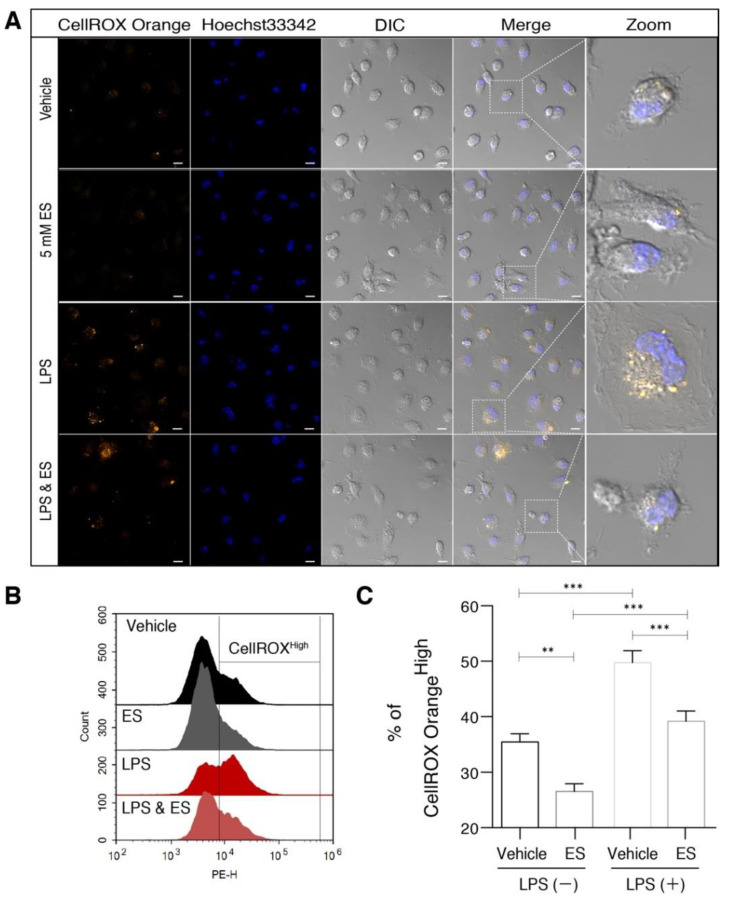
Diethyl succinate reduced ROS production in primary microglial cells. Primary microglial cells were pretreated with or without diethyl succinate (ES: 5 mM) for 3 h before being stimulated with vehicle (PBS) or LPS (100 ng/mL) for 1 h. The cells were incubated with a cellular ROS indicator, CellROX Orange, for 30 min at 37 °C. (**A**) Cells were washed and analyzed by confocal microscopy. (**B**,**C**) Representative overlay image and quantification analysis of cellular ROS by flow cytometry (*n* = 3). Scale bar = 10 µm. Data represent mean  ±  se. ** *p* < 0.01. *** *p* < 0.001.

Next, we went on to explore the mechanism by which ES decreased the levels of the M1 pro-inflammatory microglial cell population. It is well documented in the literature that microglia produce increased amounts of reactive oxygen species (ROS) as part of their inflammatory response. ROS play an important role in intracellular cell signaling, and an increase in ROS is associated with microglial activation and M1 polarization [27]. Therefore, we investigated whether the levels of the ROS were regulated by ES alone or in combination with LPS. Similar to previous reports [28], LPS treatment rapidly evoked ROS production as visualized by live-cell imaging and quantified by flow cytometry using CellROX Orange. Interestingly, ES significantly reduced the ROS production both in LPS-untreated and LPS-treated cells (Figure 2A–C). Disodium succinate exhibited no effect on the ROS levels in both the LPS-treated and untreated microglia (Figure S2).

Diethyl succinate prevented morphological transformation associated with M1 activation.

**Figure 3:**
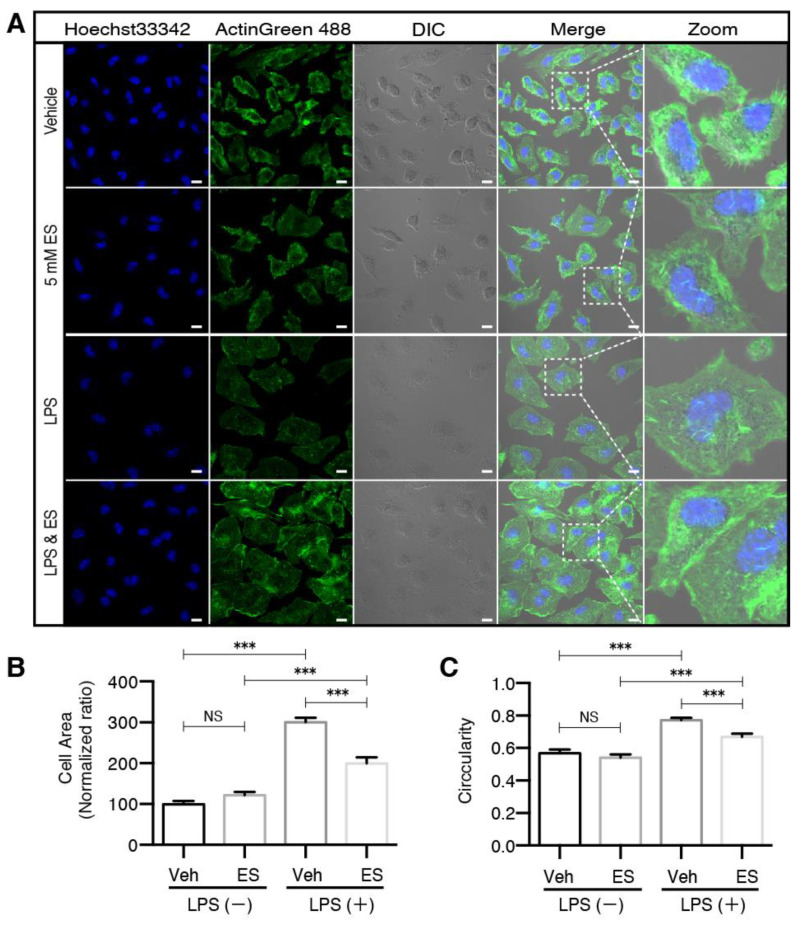
Diethyl succinate prevented morphological transformation of primary microglial cells associated with M1 activation. (**A**) Primary microglial cells were pretreated with or without diethyl succinate (ES: 5 mM) for 3 h before being stimulated with vehicle (PBS) or LPS (100 ng/mL) for 24 h. The cells were fixed and then immunocytochemically stained with ActinGreen 488, a marker for actin filaments. Nuclei were stained with Hoechst33342, and images were captured with an LSM880 confocal microscope. Scale bar = 10 µm. Quantitative analysis of (**B**) cell area and (**C**) cell circularity (proportional to cellular area or perimeter) were conducted using ImageJ software (*n* = 3 experiments). *** *p* < 0.001.

Morphological transformation of the microglia from highly ramified to round and amoeboid under pathological conditions has long been used as an indicator of microglial activation [29,30]. We examined the microglial cell morphology in response to LPS and ES treatments to further investigate the mechanisms by which diethyl succinate decreased the levels of M1 microglia. We measured two parameters, cell area and circularity, to distinguish the resting microglia from the activated microglia. In accordance with the published data [31], the microglia in a resting state were uniform in size and displayed small, round cytoplasm with an eccentric nucleus, as visualized by differential interference contrast (DIC) microscopy (Figure 3A). Contrary to this, LPS treatment caused an expansion in cytoplasmic space, which was associated with M1 polarization (Figure 3A). As we expected, diethyl succinate pretreatment significantly decreased the LPS-induced cell area expansion and circularity (Figure 3B,C), which matched the reduction of pro-inflammatory M1 phenotype microglia converted from resting microglia.

### 2.3. Diethyl Succinate Regulated Mitochondrial Fission

**Figure 4:**
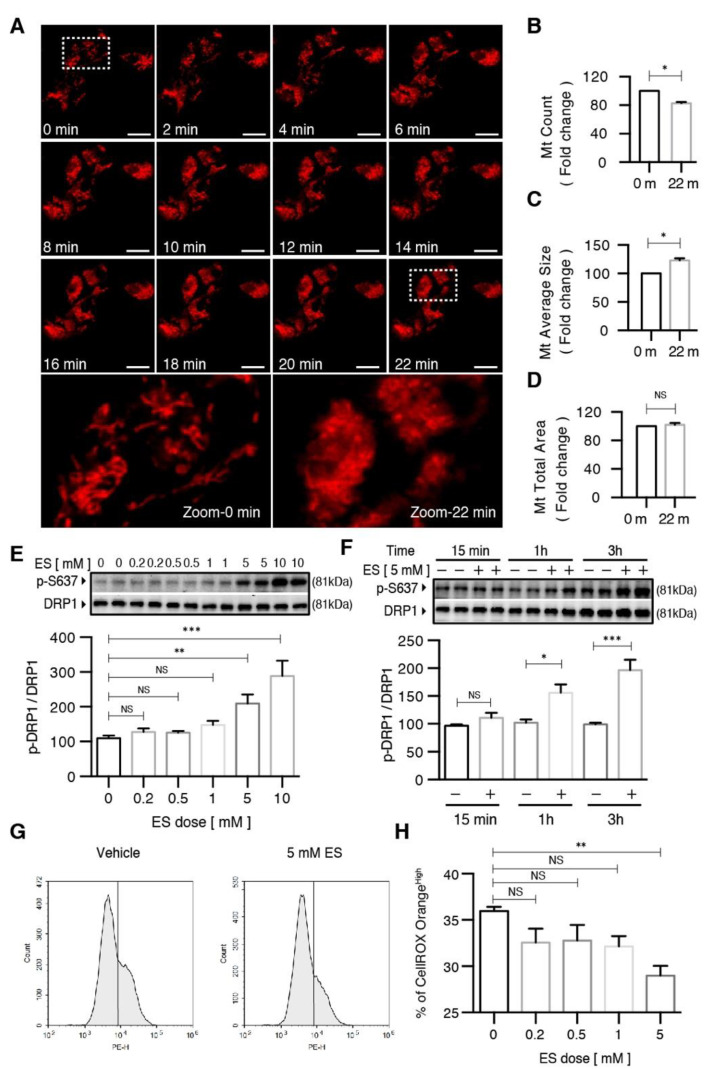
Diethyl succinate inhibited mitochondrial fission and caused a decrease in cellular ROS production in primary microglial cells. (**A**) Primary microglial cells were stained with MitoTracker Deep Red (200 nM) to localize mitochondria. After diethyl succinate (ES: 5 mM) stimulation, mitochondrial behavior in the cells was monitored using confocal microscopy (15-s intervals for 90 cycles). The lowest panel shows a higher magnification of the image in the dashed white square in the upper panel. Scale bar = 10 µm. (**B**–**D**) Quantitative analysis of mitochondria morphology in primary microglial cells treated with diethyl succinate using ImageJ software. (**E**,**F**) Primary microglial cells were treated with diethyl succinate at the indicated dose and time point. The protein levels of p-DRP1 S637 and DRP1 were determined by western blot analysis. (**G**,**H**) Primary microglial cells were treated with diethyl succinate at the indicated dose. The cells were incubated with a cellular ROS indicator, CellROX Orange, for 30 min at 37 °C. Cellular ROS levels were analyzed by flow cytometry (*n* = 3). Data represent mean  ±  se. * *p* < 0.05. ** *p* < 0.01. *** *p* < 0.001.

As a highly dynamic organelle, mitochondria constantly undergo morphological remodeling in the form of fusion and fission. Maintaining a balance between these two processes is necessary for cellular homeostasis, and tipping the scale in either direction has been linked to neurodegenerative disorders [32] and microglia in neuroinflammation [25]. To determine whether ES directly regulates this process in microglia, we tracked the mitochondrial fission in real time in response to ES treatment using time-lapse microscopy and a mitochondria-specific probe: MitoTracker Deep Red (Figure 4A). Quantitative analysis revealed that after 22 min of 5-mM ES treatment, the number of mitochondria significantly decreased (Figure 4B), and the average size of the mitochondria significantly increased (Figure 4C). Collectively, the result showed an inhibition of mitochondrial fission with the total mitochondrial area unchanged (Figure 4D).

LPS has been previously shown to promote mitochondrial fission via the activation of DRP1 in the microglial model of inflammation [25]. In addition, higher levels of DRP1 were linked to an increased production of ROS [25]. In order to determine whether the diethyl succinate-induced decrease in ROS was connected with the reduction of mitochondrial fission, we examined the protein levels of DRP1, the phosphorylation levels of DRP1 at S616 (DRP1 S617 phosphorylation enhances the recruitment of DRP1 to mitochondria and induces mitochondrial fission) and S637 (DRP1 S637 phosphorylation induces the detachment of DRP1 from mitochondria and inhibits mitochondrial fission) in microglia treated with ES at various concentrations and time points in the presence and absence of LPS.

First, we validated that LPS stimulated a continuous increase in DRP1 protein levels over a period up to 24 h (Figure S3A,B). LPS-induced DRP1 phosphorylation at S616 (p-DRP1 S616) occurred promptly at 5 min, which peaked at 15 min and started to decline after 30 min, indicating a transient increase in mitochondrial fission (Figure S3A,C). The levels of DRP1 phosphorylation at S637 (p-DRP1 S637) were not regulated by LPS (Figure S3A,D) in the microglia. Diethyl succinate did not alter the DRP1 S616 levels regardless of the presence or absence of LPS (Figure S3E,F). In contrast, a significant increase in the p-DRP1 S637 levels was observed in the microglia after diethyl succinate treatment (Figure S3E,G), suggesting a decrease in mitochondrial fission may be induced by ES. Consistent with DRP1, the protein levels of the mitochondrial fission factor (MFF) were significantly increased by LPS stimulation (Figure S3H–J). LPS did not regulate the protein levels of the mitochondrial fusion factors, mitofusin 2 (MFN2) or optic atrophy factor 1 (OPA1) in the microglia (Figure S3H,K,L).

Next, we conducted dose– and time–response experiments to determine the effect of diethyl succinate on DRP1 phosphorylation. Diethyl succinate significantly increased the levels of p-DRP1 S637 at both 5 mM and 10 mM (Figure 4E). Additionally, the levels of p-DRP1 S637 increased in a time-dependent manner in response to ES treatment (Figure 4F). Furthermore, the reduction of mitochondrial fission by diethyl succinate was accompanied by suppressed ROS levels in the primary microglial cells (Figure 4G,H).

Diethyl succinate inhibited the LPS-induced production of pro-inflammatory cytokines.

**Figure 5:**
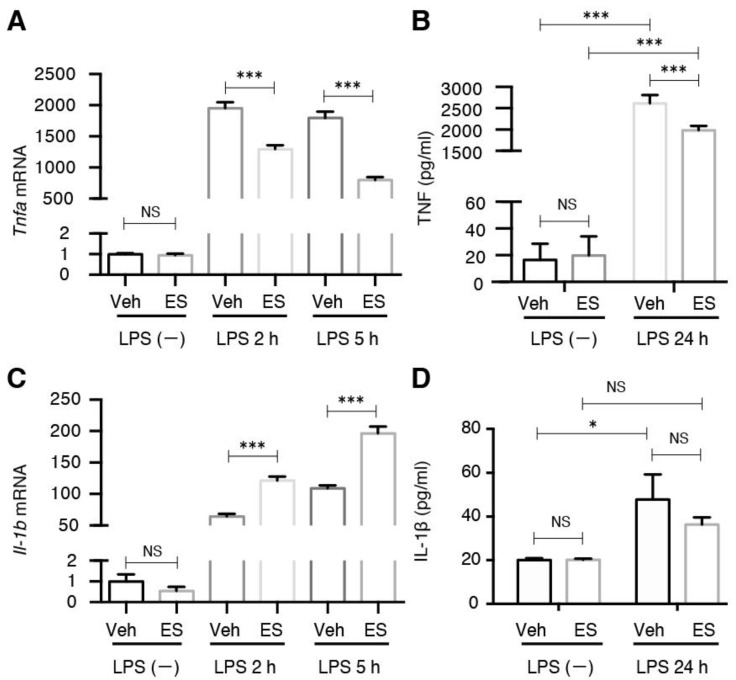
Diethyl succinate-ameliorated, LPS-induced pro-inflammatory cytokine production. (**A**) Primary microglial cells were pretreated with diethyl succinate (ES: 5 mM) for 3 h before being stimulated with vehicle (PBS) or LPS (100 ng/mL) for 2 or 5 h. Total RNA was isolated at indicated time points after LPS administration, and the gene expression level of TNF-α was determined by quantitative qPCR analysis. Each sample was normalized to β-actin expression. Gene expression levels were normalized to the vehicle-treated group. (**B**) Primary microglial cells were pretreated without or with diethyl succinate (ES: 5 mM) for 3 h before being stimulated with vehicle (PBS) or LPS (100 ng/mL) for 24 h. The culture supernatants were collected. TNF-α levels was determined using an enzyme-linked immunosorbent assay (ELISA). (**C**) Primary microglial cells were pretreated with diethyl succinate (ES: 5 mM) for 3 h before being stimulated with vehicle (PBS) or LPS (100 ng/mL) for 2 or 5 h. Total RNA was isolated at indicated time points after LPS administration, and the gene expression level of interleukin (IL)-1β was determined by qPCR analysis. Each sample was normalized to β-actin expression. Gene expression levels were normalized to the vehicle-treated group. (**D**) Primary microglial cells were pretreated without or with diethyl succinate (ES: 5 mM) for 3 h before being stimulated with vehicle (PBS) or LPS (100 ng/mL) for 24 h. The culture supernatants were then collected. IL-1β protein levels were determined by ELISA (*n* = 3). Data represent mean  ±  se. * *p* < 0.05. *** *p* < 0.001.

Microglia can be stimulated by LPS to an M1 phenotype for the expression of pro-inflammatory cytokines including tumor necrosis factor (TNF)-α and interleukin (IL)-1β. Consistent with our previous observations, the induction of TNF-α by LPS was significantly suppressed by diethyl succinate pretreatment at both the mRNA and protein levels (Figure 5A,B). Contrary to TNF-α, the induction of IL-1β by LPS was significantly enhanced by diethyl succinate pretreatment at the mRNA level but not changed at the protein level (Figure 5C,D).

### 2.4. The Effects of Diethyl Succinate on Mitochondrial Fission and ROS Production Are Receptor-Independent

**Figure 6:**
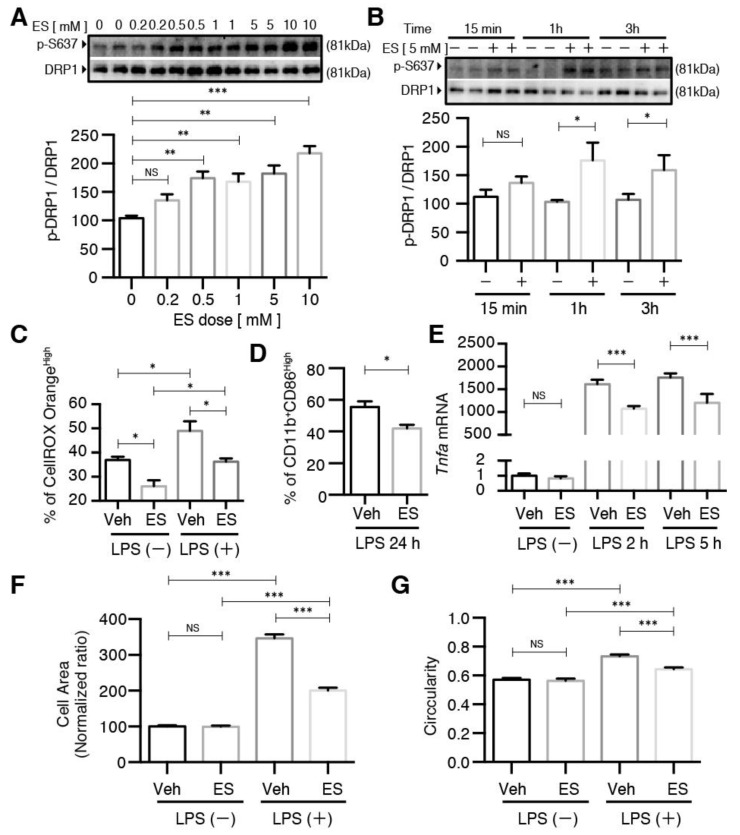
The effects of diethyl succinate on primary microglial cells was receptor-independent. (**A**,**B**) Primary microglial cells from *Sucnr1*−/− mice were treated with diethyl succinate at the indicated doses and time points. The protein levels of p-DRP1 S637 and DRP1 were determined by western blot analysis. (**C**–**G**) Primary microglial cells from *Sucnr1*−/− mice were pretreated with or without diethyl succinate (ES: 5 mM) for 3 h before being stimulated with vehicle (PBS) or LPS (100 ng/mL) for the indicated time. (**C**) The cells were incubated with a cellular ROS indicator, CellROX Orange, for 30 min at 37 °C. Cellular ROS levels were analyzed by flow cytometry. (**D**) Cells were washed and analyzed for flow cytometry. (**E**) Total RNA was isolated at indicated time points after LPS administration, and the gene expression level of *Tnfa* was determined by qPCR analysis and normalized to β-actin expression in each sample. Gene expression levels in each group normalized to the vehicle-treated group are shown. (**F**,**G**) The cells were fixed and then immunocytochemically stained for ActinGreen 488, and quantitative analysis of the cell area and cell circularity were performed using ImageJ Software (*n* = 3). * *p* < 0.05. ** *p* < 0.01. *** *p* < 0.001.

The unique effects of the membrane-permeable diethyl succinate, but not the disodium succinate, on mitochondrial fission and ROS production suggest a protective role of intracellular succinate in the primary microglial cells. In other words, such effects should be succinate receptor-independent. We isolated the primary microglial cells from *Sucnr1*−/− mice and subjected them to the same experiments we performed with wild-type microglia. Indeed, diethyl succinate significantly increased the p-DRP1 S637 levels in the *Sucnr1*−/− cells (Figure 6A). It is notable that the *Sucnr1*−/− cells responded to a much lower concentration of diethyl succinate than the wild-type microglia (Figure 4C). It is possible that part of the cell-permeable diethyl succinate still bound to SUCNR1 in the WT cells, and therefore a higher concentration was required for the WT cells to achieve a similar intracellular effect in *Sucnr1*−/− microglial cells. The phosphorylation levels of DRP1 S637 were also significantly increased at 1 h and 3 h after diethyl succinate treatment in *Sucnr1*−/− cells, similar to the wild-type cells (Figure 4D and Figure 6B). Furthermore, diethyl succinate significantly reduced ROS production in both the LPS-untreated and LPS-treated *Sucnr1*−/− cells, indicating that the succinate receptor was not involved in the protection mechanism exerted by intracellular succinate (Figure 6C). M1 polarization in the LPS-treated *Sucnr1*−/− cells was significantly decreased by pretreatment with diethyl succinate (Figure 6D). Consistent with these observations, the *TNF-α* mRNA levels were significantly decreased by diethyl succinate pretreatment in the LPS-stimulated *Sucnr1*−/− primary microglial cells (Figure 6E). The increases in the *Sucnr1*−/− cells’ size and circularity by LPS stimulation were diminished in the microglial cells pretreated with ES for 3 h (Figure 6F,G and Figure S4). Similarly, ES prevented the polarization toward the M1 phenotype in the *Sucnr1*−/− microglia, as it did in the WT cells.

These data indicate that receptor-independent intracellular succinate signaling may counteract LPS-induced activation of microglia. Of note, such counteraction seemed to occur mainly through regulation of the mitochondria and ROS levels through DRP1 phosphorylation. The LPS-induced inflammatory response in the microglia was mediated by the ERK and p38 MAPK pathways (Figure S5A–C), leading to the expression of pro-inflammatory cytokines such as TNF-α and IL-6. This expression profile is concurrent with adopting the M1 phenotype [33]. However, pretreatment with diethyl succinate did not alter the levels of phosphorylated ERK1/2 or P38 MAPK induced by LPS (Figure S5D–F).

## 3. Discussion

Our study adds to the existing understanding of the link between succinate accumulation, mitochondrial dynamics and microglial activation in response to LPS-induced inflammation in the primary microglia. We demonstrated that pretreatment with cell-permeable diethyl succinate prevented the conversion of a subset of resting microglia into the pro-inflammatory M1 phenotype, thus protecting these cells from cytotoxicity associated with the secretion of inflammatory cytokines. We also showed that succinate’s protective role was related to modulating mitochondrial dynamics, specifically by inhibiting fission. Additionally, we found that diethyl succinate reduced ROS production in the primary microglia exposed to LPS. This is the first study to demonstrate that diethyl succinate counteracts the inflammatory responses induced by LPS in microglia, suggesting a potent anti-inflammatory role of succinate independent of its extracellular receptor.

Considering that previous studies have associated succinate with increased inflammatory response [14,15,16,18,21], this calls for extra caution in analyzing and interpreting our results on the microglial cells. First, microglia are a unique cell type [34,35], despite sharing the same progenitors with the macrophages and dendritic cells which were used in the previous studies. Secondly, most of the studies demonstrated the extracellular role of succinate through activating its receptor while leaving the intracellular role of succinate unaddressed. Third, we conducted the experiments by pretreating the microglia with diethyl succinate prior to LPS exposure. This allowed us to dissect the impact of increased intracellular succinate on microglia activation from the effects of intracellular succinate increases due to other stimuli. such as LPS or hypoxia.

Previous reports indicated a link between abnormal mitochondrial fission and LPS-induced microglial activation [22,36]. It was shown that the treatment of microglia with LPS increased mitochondrial fission through promoting phosphorylation of DRP1 at Ser616. This led to an increase in the levels of ROS, which in turn activated MAPK and NFkB and caused the expression of pro-inflammatory cytokines. Conversely, the inhibition of mitochondrial fission by Mdivi1 reduced MAPK and NFkb, resulting in a decrease in the levels of pro-inflammatory cytokines and thus inhibiting microglial activation. Our study provides an alternative mechanism, suggesting that LPS-induced elevation of intracellular succinate may impact the microglial activation status by directly modulating the mitochondrial dynamics. Mitochondrial fission is a tightly controlled process regulated by a number of proteins. DRP1 is a major pro-fission protein that resides in the cytosol, but upon being phosphorylated at Ser616, it is translocated to the mitochondria, where it forms helical oligomers wrapping around the mitochondria to constrict and finally scission them into smaller fragments [37,38].

DRP1 can also be phosphorylated Ser637, which favors its detachment from the mitochondria and decreases fission [39,40]. Our study showed that diethyl succinate significantly increased levels of DRP1 phosphorylated at Ser637 at 1 h of treatment (Figure 4F). This was concomitant with a significant decrease in ROS at the same time point of 1 h (Figure 2A,C), indicating that diethyl succinate on its own inhibited mitochondrial fission. When the microglia were pretreated with diethyl succinate for 3 h followed by 10 min of LPS, we observed increases in both S616 and S637. This suggests that LPS and diethyl succinate may evoke opposing effects on fission and fusion, and the timing of succinate and LPS administration could be pivotal to the overall outcome. As a consequence, the combined effects of LPS and ES pretreatment led to the release of lower levels of ROS compared with those elicited by LPS alone. This may have caused the observed decrease in the expression of pro-inflammatory factors, the levels of which were not high enough to ensure the conversion of a subset of the resting microglia into the M1 pro-inflammatory ones and hence the significant decrease in the population of M1 microglia observed in Figure 1.

Chronic neuroinflammation is a common hallmark in diseases such as Alzheimer’s disease (AD) and Parkinson’s disease (PD) among many others. Abnormal activation of microglia has been implicated in contributing to a chronic inflammatory status. Our study indicates an anti-inflammatory role of diethyl succinate, countering LPS-induced inflammation in a primary microglial cell model. This is an exciting discovery for the potential of the endogenous succinate as an anti-inflammatory molecule, in contrast to the action of extracellular succinate through its receptor. Our study suggests that it may be critical to retain the beneficial effect of intracellular succinate while controlling its releasing out of cytosol and the initiation of SUCNR1-mediated pro-inflammatory responses. Further studies focusing on the regulation of the succinate production and transportation are essential to explore the strategy of utilizing succinate in the control of chronic neuroinflammation.

## 4. Materials and Methods

The reagents included disodium succinate (catalog number: 14160), diethyl succinate (catalog number: 112402) and lipopolysaccharide (catalog number: L2637, St. Louis, MO, USA) from Millipore Sigma (St Louis, MO, USA). Phosphate buffered saline (catalog number: 14190250), high glucose Dulbecco’s Modified Eagle Medium (catalog number: 11995-065), Penicillin Streptomycin (catalog number: 15140122) and fetal bovine serum (catalog number: 16000-069) were obtained from Thermo Fisher Scientific (Hampton, NH, USA).

As for the animals, C57B/6 strain WT and *Sucnr1*−/− mice were derived from *Sucnr1*+/− breeders. All animal experiments related to this study were approved by the Institutional Animal Care and Use Committee (IACUC) under protocol numbers 160108 and 170405 and performed in accordance with the Division of Laboratory Animal Resources (DLAR). All the animals were housed in specific pathogen-free units at the New York University animal facility.

For the mouse microglial cell culture, the primary microglia culture was performed as previously described [41]. Briefly, brains of postnatal mice within 1 to 2-day-old were dissected, stripped of their meninges, mechanically dissociated by pipetting into a single-cell suspension and centrifuged at 1000 RPM for 5 min. The cell pellet was washed once and resuspended in high glucose Dulbecco’s Modified Eagle Medium supplemented with 100 IU/mL penicillin, 100 μg/mL streptomycin and 10% fetal bovine serum. After being filtered through a 70-μm cell strainer (BD Falcon, Bedford, MA, USA), the cells were seeded in a 10-cm culture dish and maintained at 37 °C in a humidified incubator with 5% CO_2_. At the 3rd and 10th day, the culture medium was changed. After 15 days in vitro, the mixed glia cultures were dissociated by trypsinization, and the cell suspension was seeded on a petri dish and incubated for 30 min in a CO_2_ incubator. The media were then removed, and the adherent cells were washed twice and harvested as the primary microglia. The microglia were reseeded in culture plates. The purity of the microglia was greater than 95%, as determined by flow cytometry using PE anti-mouse/human CD11b antibody.

Concerning flow cytometry, the primary microglial cells were gently collected using a cell scraper (catalog number: 353085, Coring, New York, NY, USA). The cells were pelleted by centrifugation (1000 RPM, 5 min) and resuspended in a cell-staining buffer (catalog number: 420201, Biolegend, San Diego, CA, USA). The cells were stained with the appropriate antibodies (Supplementary Table S1) for 30 min on ice, washed and analyzed using a CytoFLEX flow cytometer (Beckman Coulter, Indianapolis, IN, USA) and its CytExpert software. For oxidative stress detection, the cells were stained using CellROX Orange Reagent (catalog number: C10443, Thermo Fisher Scientific, Hampton, NH, USA) according to the manufacturer’s instructions.

Regarding total RNA isolation and real time-PCR, the total RNA was isolated from the primary microglial cells using an RNeasy Plus Mini Kit (catalog number: 74136, Qiagen, Venlo, Netherlands). RNA (500 ng) was converted into cDNA using a reverse transcriptase kit (catalogue number: N8080234, Thermo Fisher Scientific, Hampton, NH, USA) according to the manufacturer’s instructions. The cDNA was used for quantitative real-time PCR using the power 2 × SYBR Green PCR Master Mix (catalog number: 4309155, Applied Biosystems, Warrington, UK) and monitored on a CFX384 Touch Real-Time PCR Detection System (Bio-Rad, Philadelphia, PA, USA). The primer sequences of the selected genes are provided in Supplementary Table S2. Relative gene expression versus the control was normalized to β-actin gene expression.

For western blot analysis, we extracted the total protein using the RIPA lysis buffer (catalogue number: 89900, Thermo Fisher Scientific, Hampton, NH, USA) and used a Pierce BCA Protein Assay Kit (catalogue number: 23225, Thermo Fisher Scientific, Hampton, NH, USA) to determine the protein concentrations. The protein samples were denatured in Laemmli SDS sample buffer (catalogue number: J60660, Alfa Aesar, Tewksbury, MA, USA) supplemented with NuPAGE Sample-Reducing Agent (catalog number: NP0009, Thermo Fisher Scientific, Hampton, NH, USA) and loaded into a 10% SDS–polyacrylamide gel electrophoresis gel (catalog number: XP00105BOX, Thermo Fisher Scientific, Hampton, NH, USA). The gel was preceded according to a standard polyvinylidene difluoride membrane transfer, incubated with primary and secondary antibodies (Supplementary Table S1) and visualized using the peroxidase substrate SuperSignal West Dura (catalog number: 34076, Thermo Fisher Scientific, Hampton, NH, USA) for enhanced chemiluminescence on the ChemiDoc XRS System (BioRad Laboratories, Inc., Hercules, CA, USA).

For immunohistochemistry and live-cell imaging, the primary microglial cells were seeded into an 8-well glass chamber slide (catalog number: PEZGS0816, Merck Millipore, Danvers, MA, USA) and fixed by immersion in 4% formaldehyde overnight at 4 °C. Then, the cells were stained by using ActinGreen 488 ReadyProbes Reagent (catalog number: R37110, ThermoFisher Scientific, Waltham, MA, USA) according to the manufacturer’s instructions. For live-cell imaging, the cells were seeded into 35-mm Nunc glass-bottom dishes (catalog number: 150682, ThermoFisher Scientific) and preloaded with Mito-tracker DeepRed, Hoechst33342 and CellROX Orange, and images were captured with a Zeiss LSM880 confocal microscope with Zeiss ZEN software using a 60X oil immersion objective lens. Image analysis and quantification was performed in ImageJ and ZEN software (Carl Zeiss AG, Oberkochen, Germany).

Regarding, ELISA, the TNF-α protein expression levels in the conditioned media were determined by an enzyme-linked immunosorbent assay. The assay was performed according to TNF-α ELISA Kit protocols (catalog number: MTA00B, R&D System, Minneapolis, MN, USA) and IL-1β ELISA Kit protocols (catalog number: MLB00C, R&D System, Minneapolis, MN, USA). A SpectraMax M5 microplate reader (Molecular Devices, San Jose, CA, USA) was used to measure absorbance at 450 nm.

As for statistical analysis, all experiments were conducted with 3 biological samples and repeated at least 3 times. All of the statistical analysis was performed using GraphPad Prism 6.0 software (GraphPad; San Diego, CA, USA). As indicated in the figure legends, all values in the figures and text are expressed as means ± standard error (se), and an unpaired, two-tailed Student’s *t*-test was used for comparison of two independent variables, while one-way ANOVA was used for comparisons of one independent variable to compare three or more groups, followed by Tukey’s post hoc test for multiple comparisons. Then, two-way ANOVA was performed where two independent variables were considered, followed by Tukey’s post hoc test for multiple comparisons. Significance levels were set at *p* < 0.05, *p* < 0.01 and *p* < 0.001.

## Data Availability

All raw images of the results will be uploaded to public domain.

## Supplementary Materials

The following are available online at https://www.mdpi.com/article/10.3390/metabo11120854/s1. Figure S1. Disodium succinate did not alter M1 population in primary microglial cells. Primary microglial cells were pretreated without or with disodium succinate (ES: 5 mM) for 3 h before being stimulated with vehicle (PBS) or LPS (100 ng/mL) for 24 h. Cells were washed and analyzed for flow cytometry. (A,B) Representative overlay image and quantification analysis of M1 surface marker. (C,D) Representative overlay image and quantification analysis of M2 surface marker (*n* = 3). Data represent mean  ±  se. *** *p* < 0.001. Figure S2. Disodium succinate did not reduce ROS production in primary microglial cells. Primary microglial cells were pretreated without or with disodium succinate (ES: 5 mM) for 3 h before being stimulated with vehicle (PBS) or LPS (100 ng/mL) for 1 h. The cells were incubated with a cellular ROS indicator, CellROX Orange, for 30 min at 37 °C. Cells were washed and analyzed by flow cytometry. (A) Representative overlay image of cellular ROS. (B) Quantification analysis of cellular ROS by flow cytometry (*n* = 3). Data represent mean  ±  se. *** *p* < 0.001. Figure S3. Diethyl succinate promoted phosphorylation of DRP1 at Ser-637 in primary microglial cells. (A–D) Primary microglial cells were treated with LPS (100 ng/mL) at indicated time point. The protein levels of p-DRP1 S616, p-DRP1 S637, DRP1 and tubulin were determined by western blot analysis. (E–G) Primary microglial cells were pretreated without or with diethyl succinate (ES: 5 mM) for 3 h before being stimulated with vehicle (PBS) or LPS (100 ng/mL) for 10 min. The protein levels of p-DRP1 S616, p-DRP1 S637 and DRP1 were determined by western blot analysis. (H–L) Primary microglial cells were pretreated without or with disodium succinate (SS: 5 mM) or diethyl succinate (ES: 5 mM) for 3 h before being stimulated with LPS (100 ng/mL) for 24 h. The protein levels of DRP1, MFF, MFN2, OPA1 and ACTIN were determined by western blot analysis. (*n* = 3) Data represent mean  ±  se. * *p* < 0.05. ** *p* < 0.01. *** *p* < 0.001. Figure S4. Diethyl succinate prevented morphological transformation of Sucnr1−/− primary microglial cells associated with M1 activation. (A) Primary Sucnr1−/− microglial cells were pretreated without or with diethyl succinate (ES: 5 mM) for 3 h before being stimulated with vehicle (PBS) or LPS (100 ng/mL) for 24 h. The cells were fixed and then immunocytochemically stained for ActinGreen 488, a marker for actin filaments. Nuclei were stained with hoechst33342, and images were captured with an LSM880 confocal microscope. Scale bar = 10 µm. Figure S5. Diethyl succinate did not affect lipopolysaccharide-induced MAPK activation in primary microglial cells. (A–C) Primary microglial cells were treated with LPS (100 ng/mL) at indicated time point. The protein levels of p-ERK1/2, ERK1/2, p-P38 and P38 were determined by western blot analysis. (D–F) Primary microglial cells were pretreated without or with diethyl succinate (ES: 5 mM) for 3 h before being stimulated with vehicle (PBS) or LPS (100 ng/mL) for 10 min. The protein levels of p-ERK1/2, ERK1/2, p-P38 and P38 were determined by western blot analysis (*n* = 3). Data represent mean  ±  se. ** *p* < 0.01. *** *p* < 0.001. Table S1. Antibodies used in this study. Table S2. Primers used in this study.
