# Supplementary material for: Diethyl Succinate Modulates Microglial Polarization and Activation by Reducing Mitochondrial Fission and Cellular ROS
Source: Metabolites. 2021 Dec 8;11(12):854. doi: 10.3390/metabo11120854 (PMC8705220; doi:10.3390/metabo11120854)
Supplement: Supplementary file 1 [file metabolites-11-00854-s001.zip › metabolites-1477629-supplementary.pdf]

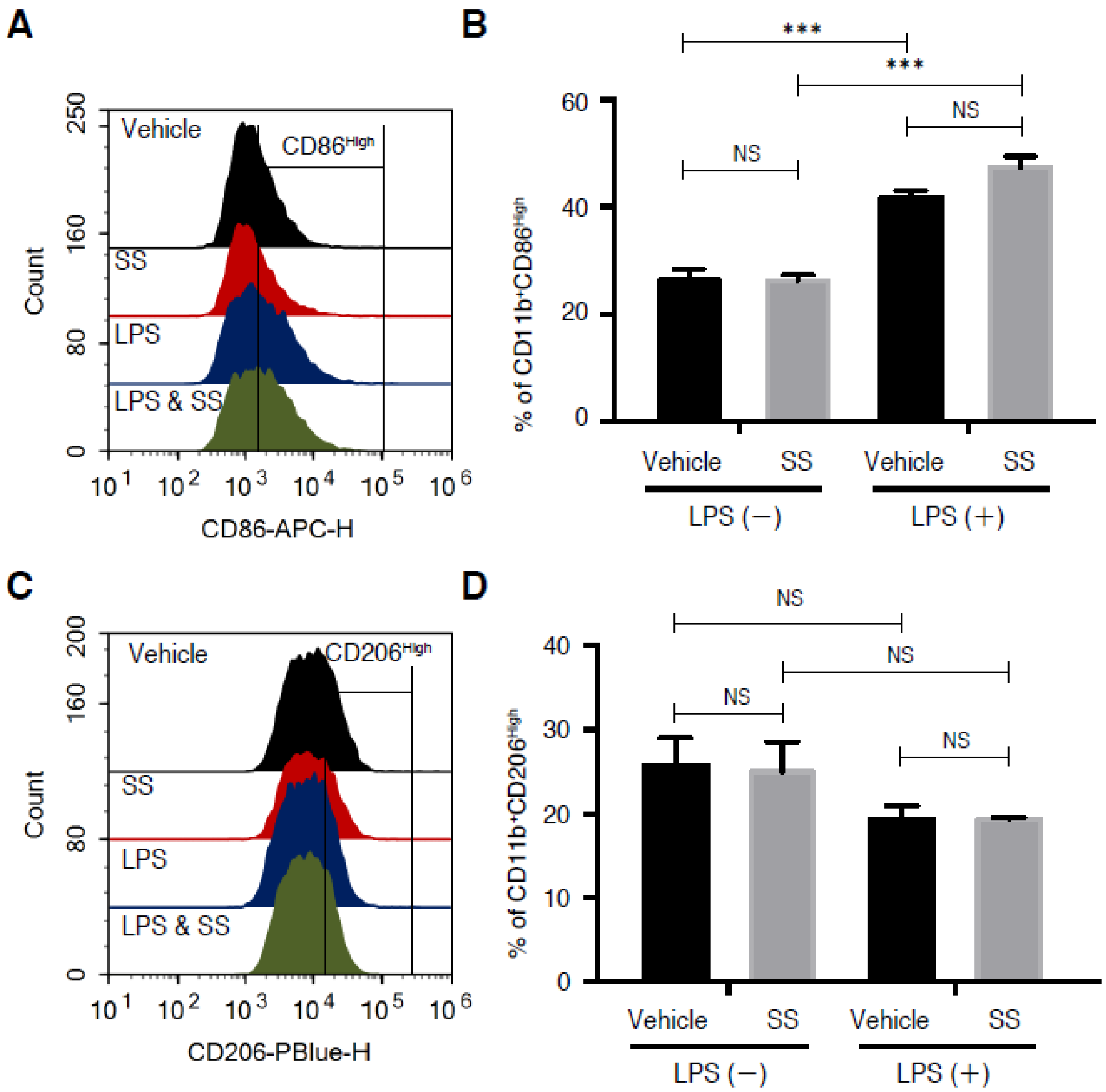

Figure S1. Disodium succinate did not alter M1 population in primary microglial cells. Primary microglial cells were pretreated without or with disodium succinate (ES: 5 mM) for 3 h before being stimu-lated with vehicle (PBS) or LPS (100 ng/mL) for 24 h. Cells were washed and analyzed for flow cytometry. (**A**,**B**) Representative overlay image and quantification analysis of M1 surface marker. (**C**,**D**) Representative overlay image and quantification analysis of M2 surface marker (n = 3). Da-ta represent mean ± se. *** $p < 0.001$.

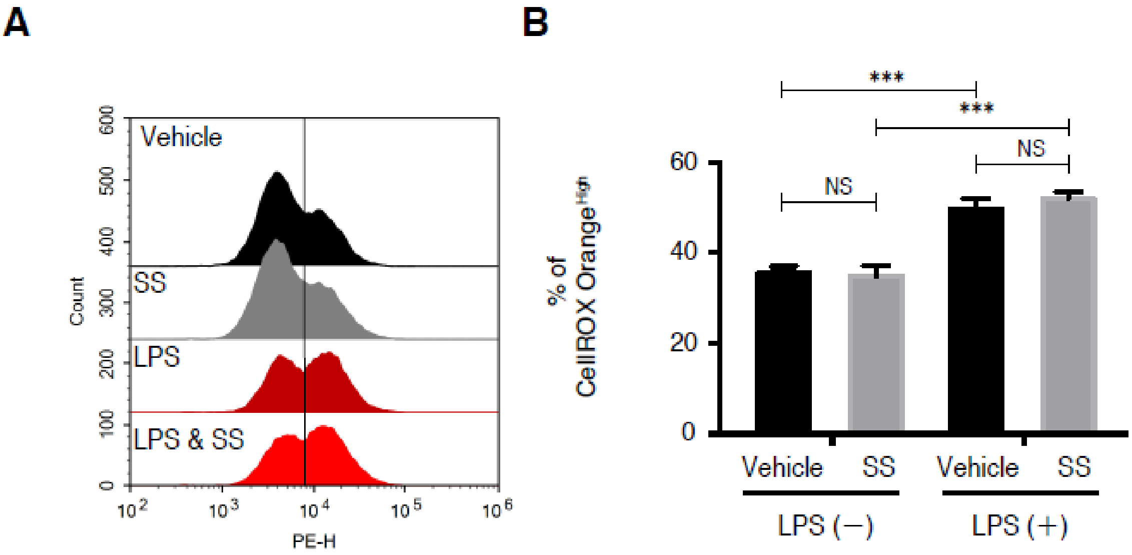

Figure S2. Disodium succinate did not reduce ROS produc-tion in primary microglial cells. Primary microglial cells were pretreated without or with disodium succinate (ES: 5 mM) for 3 h before being stimulated with vehicle (PBS) or LPS (100 ng/mL) for 1 h. The cells were incubated with a cellular ROS indicator, CellROX Orange, for 30 min at 37 °C. Cells were washed and analyzed by flow cytometry. (**A**) Representative overlay image of cellular ROS. (**B**) Quantification analysis of cellular ROS by flow cytometry ($n = 3$). Data represent mean ± se. *** $p < 0.001$.

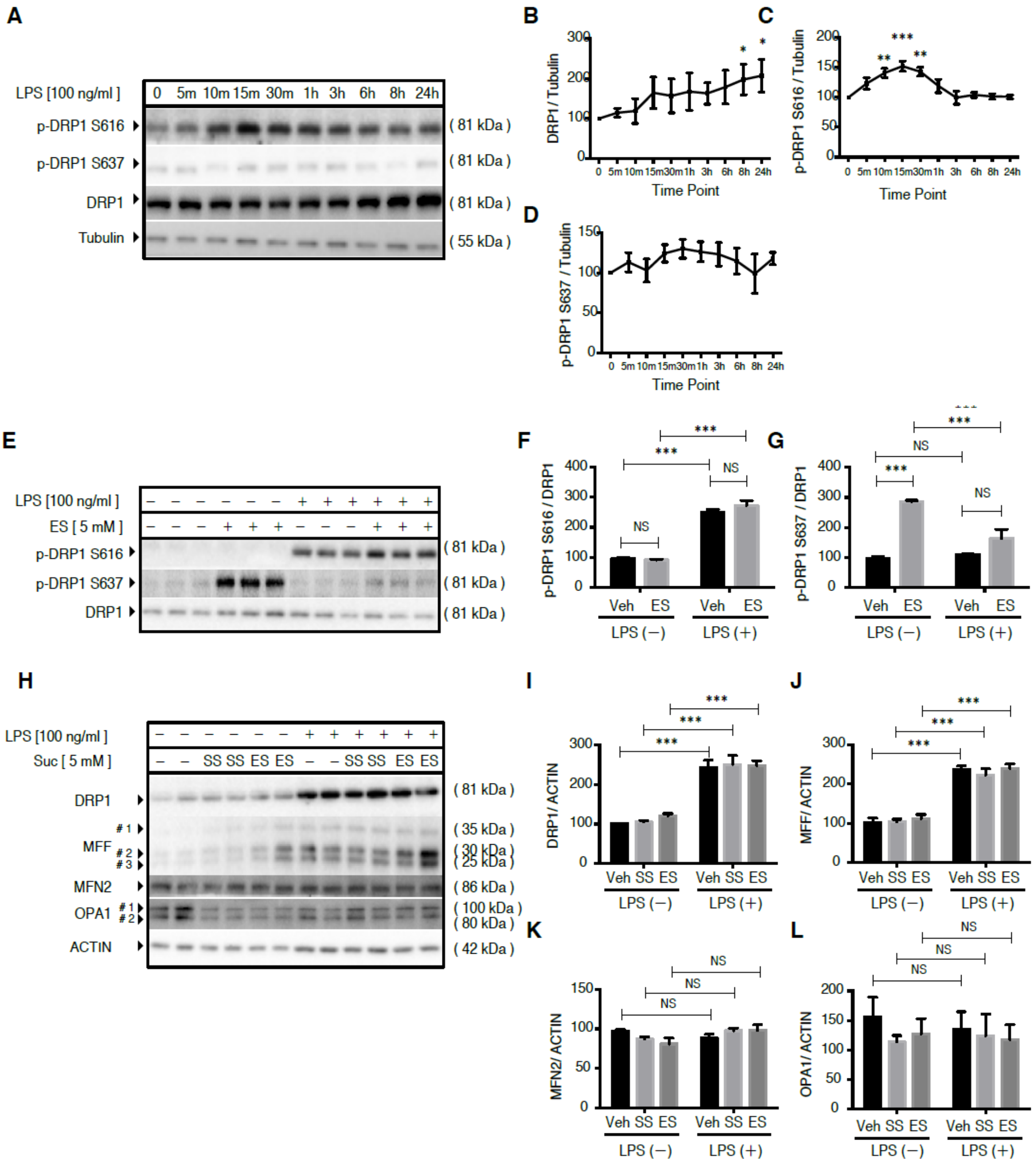

Figure S3. Diethyl succinate promoted phosphorylation of DRP1 at Ser-637 in primary microglial cells. (**A–D**) Primary microglial cells were treated with LPS (100 ng/mL) at in-dicated time point. The protein levels of p-DRP1 S616, p-DRP1 S637, DRP1 and tubulin were de-termined by western blot analysis. **(E–G**) Primary microglial cells were pretreated without or with diethyl succinate (ES: 5 mM) for 3 h before being stimulated with vehicle (PBS) or LPS (100 ng/mL) for 10 min. The protein levels of p-DRP1 S616, p-DRP1 S637 and DRP1 were determined by western blot analysis. (**H–L**) Primary microglial cells were pretreated without or with disodium succinate (SS: 5 mM) or diethyl succinate (ES: 5 mM) for 3 h before being stimulated with LPS (100 ng/mL) for 24 h. The protein levels of DRP1, MFF, MFN2, OPA1 and ACTIN were deter-mined by western blot analysis. (n = 3) Data represent mean ± se. * $p < 0.05$. ** $p < 0.01$. *** $p < 0.001$.

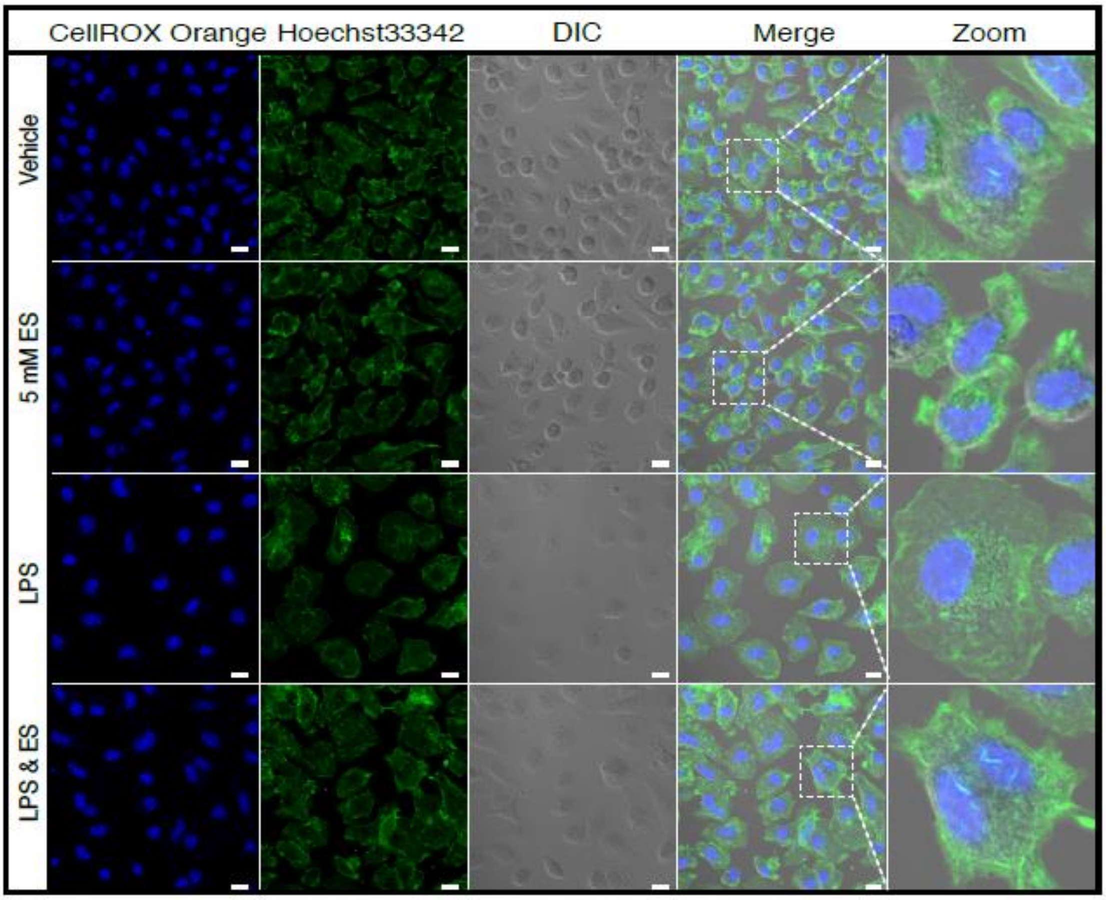

Figure S4. Diethyl succinate prevented morphological transformation of *Sucnr1*−/− primary microglial cells associated with M1 activation. (A) Primary *Sucnr1*−/− microglial cells were pre-treated without or with diethyl succinate (ES: 5 mM) for 3 h before being stimulated with vehicle (PBS) or LPS (100 ng/mL) for 24 h. The cells were fixed and then immunocytochemically stained for ActinGreen 488, a marker for actin filaments. Nuclei were stained with hoechst33342, and images were captured with an LSM880 confocal microscope. Scale bar = 10 μm.

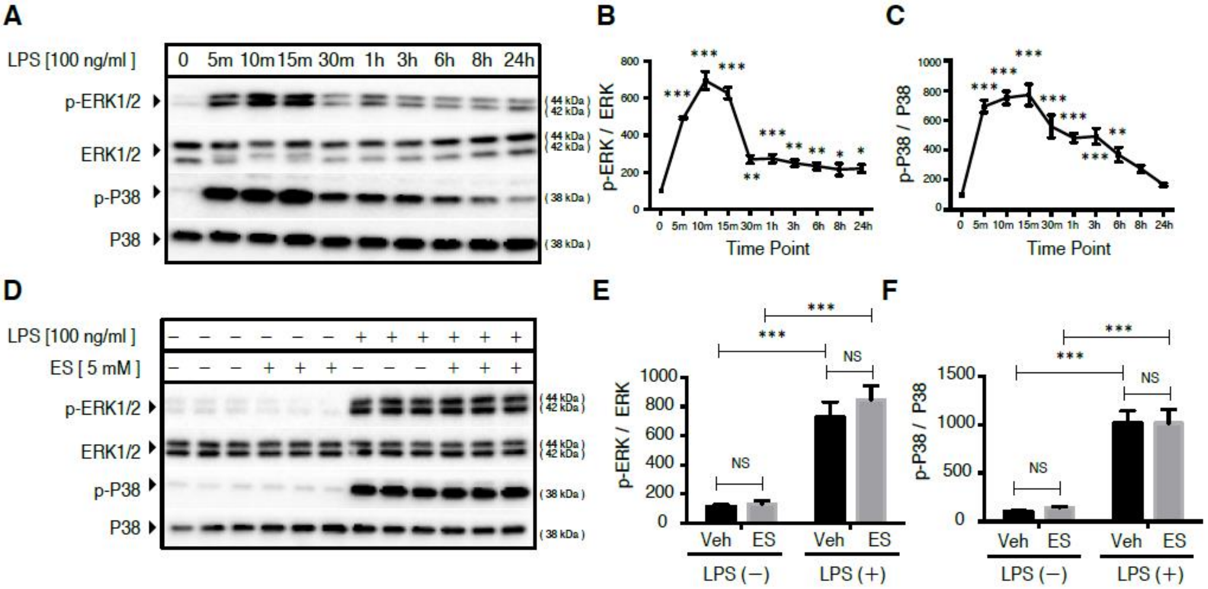

Figure S5. Diethyl succinate did not affect lipopolysaccharide-induced MAPK activation in primary microglial cells. (**A–C**) Primary microglial cells were treated with LPS (100 ng/mL) at indicated time point. The protein levels of p-ERK1/2, ERK1/2, p-P38 and P38 were determined by western blot analysis. (**D–F**) Primary microglial cells were pretreated without or with diethyl succinate (ES: 5 mM) for 3 h before being stimulated with vehicle (PBS) or LPS (100 ng/mL) for 10 min. The protein levels of p-ERK1/2, ERK1/2, p-P38 and P38 were determined by western blot analysis (n = 3). Data represent mean ± se. ** $p < 0.01$. *** $p < 0.001$..

Table S1. Antibodies used in this study.

Antibodies used for Flow Cytometry
PE anti-Mouse/Human CD11b (Cat # 101208; 1:100 dilution ; BioLegend, San Diego, CA, USA)
APC anti-Mouse CD86 (Cat # 105012; 1:100 dilution ; BioLegend, San Diego, CA, USA)
Brilliant Violet 421™ anti-mouse CD206 (Cat # 141717; 1:100 dilution ; BioLegend, San Diego, CA, USA)
Antibodies used for Western Blot
Phospho-DRP1 (Ser637) Antibody (Cat # 4867s; 1:1000 dilution; Cell signaling, Danvers, MA, USA)
Phospho-DRP1 (Ser616) Antibody (Cat # 3455s; 1:1000 dilution; Cell signaling, Danvers, MA, USA)
Purified Mouse Anti-DLP1 (Cat # 611113; 1:1000 dilution ; BD Biosciences, Sparks, MD, USA)
Mouse monoclonal anti-OPA1 (Cat # 612606; 1:1000 dilution ; BD Biosciences, Sparks, MD, USA)
MFN2 monoclonal antibody (Cat # H00009927-M03; 1:1000 dilution ; Abnova, Taipei, Taiwan)
Mff antibody (Cat # 17090-1-AP; 1:1000 dilution ; Proteintech, Rosemont, IL, USA)
Rabbit polyclonal anti-phospho-p38 MAPK (Thr180/Tyr182) (Cat # 4511s; 1:1000 dilution; Cell signaling, Danvers, MA, USA)
Rabbit polyclonal anti-p38 MAPK (Cat # 8690; 1:1000 dilution; Cell signaling, Danvers, MA, USA)
Rabbit monoclonal anti-phospho-ERK1/2 (Thr202/Tyr204) (Cat # 4370s; 1:1000 dilution; Cell signaling, Danvers, MA, USA)
Rabbit monoclonal anti-ERK1/2(137F5) (Cat # 4695s; 1:1000 dilution; Cell signaling, Danvers, MA, USA)
β-Actin (13E5) Rabbit mAb (Cat # 4970s; 1:1000 dilution; Cell signaling, Danvers, MA, USA)
α-Tubulin (11H10) Rabbit mAb (Cat # 2125s; 1:1000 dilution; Cell signaling, Danvers, MA, USA)
Horse anti-mouse IgG, HRP-linked antibody (1:5000 dilution; Cell signaling, Danvers, MA, USA)
Goat anti-rabbit IgG, HRP-linked antibody (1:5000 dilution; Cell signaling, Danvers, MA, USA)

Table S2. Real-time PCR primers used in this study.

Gene	Forward primer (5' – 3')	Reverse primer (5' – 3')
Cd86	AGCACGGACTTGAACAACCAGAC	TTGTAAATGGGCACGGCAGATA
Cd206	CTGATGCAAACCACACATGCAC	TTCAGGCCTCAATCCAACCAA
Tnfa	ATCCGCGACGTGGAACTG	ACCGCCTGGAGTTCTGGAA
Il-1b	GGAGAACCAAGCAACGACAAAATA	TGGGGAACTCTGCAGACTCAAAC
Actin	TCATCACTATTGGCAACGACG	AACAGTCCGCCTAGAAGCAC
